# Hydrogel-Based Controlled Release of Phytohormones: Improving Bioavailability and Early Plant Development Outcomes

**DOI:** 10.3390/ijms27125221

**Published:** 2026-06-09

**Authors:** Ana V. Torres-Figueroa, Andrés Ochoa-Meza, Lidianys M. Lewis-Lujan, Simon B. Iloki-Assanga, Cinthia J. Pérez-Martínez, Dora E. Rodríguez-Félix, Sergio F. Moreno-Salazar, Teresa del Castillo-Castro, Sergio de los Santos-Villalobos

**Affiliations:** 1 Departamento de Ciencias Químico Biológicas, Universidad de Sonora, Hermosillo 83000, Mexico; lidianys.lewis@unison.mx (L.M.L.-L.); simon.iloki@unison.mx (S.B.I.-A.); jhovanna.perez@unison.mx (C.J.P.-M.); 2 Departamento de Agricultura y Ganadería, Universidad de Sonora, Carr. Bahía de Kino, Km. 21. Apartado Postal 305, Hermosillo 83323, Mexico; andres.ochoa@unison.mx (A.O.-M.); sergio.moreno@unison.mx (S.F.M.-S.); 3 Departamento de Investigación en Polímeros y Materiales, Universidad de Sonora, Hermosillo 83000, Mexico; dora.rodriguez@unison.mx (D.E.R.-F.); teresa.delcastillo@unison.mx (T.d.C.-C.); 4 Laboratorio de Biotecnología del Recurso Microbiano, Departamento de Ciencias Agronómicas y Veterinarias, Instituto Tecnológico de Sonora, 5 de Febrero 818 Sur, Colonia Centro, Ciudad Obregón 85000, Mexico

**Keywords:** early plant development, controlled release systems, plant-derived bioactives, functional plant biochemistry, phytohormone signaling

## Abstract

The application of hydrogels in agriculture has gained increasing attention for its potential to support early plant development, a stage highly sensitive to environmental and biochemical fluctuations. This review examines the role of hydrogel-mediated delivery of plant-derived bioactives, particularly phytohormones, in regulating their availability during seed germination and seedling establishment. Evidence from recent studies shows that hydrogels function as three-dimensional polymeric matrices that enhance water retention and provide controlled delivery of encapsulated phytohormones. These properties are consistently associated with improved germination, root development, stress tolerance, and early plant establishment. Importantly, hydrogel-based systems regulate the timing, localization, and duration of phytohormone exposure, contributing to improved developmental responses while reducing losses and phytotoxic effects associated with conventional applications. Overall, this work highlights the need for further field-scale studies to determine how controlled release strategies can be leveraged to optimize plant development under realistic agricultural conditions.

## 1. Introduction

Climate change and the intensification of anthropogenic activities have markedly increased the frequency and severity of abiotic and biotic stresses in agricultural systems, posing a substantial threat to global crop productivity and food security [1,2]. These pressures are particularly critical during the earliest stages of plant development, when seed germination, seedling establishment, and initial root system formation are highly sensitive to environmental fluctuations [3]. During this phase, plants depend on tightly coordinated biochemical responses involving signal perception, transduction, and metabolic regulation to ensure successful establishment and survival [4]. However, the limited physiological resilience at these stages often makes early development a rate-limiting stage for overall crop performance under stress conditions [3].

Within this context, plant-derived bioactives, particularly phytohormones, play a central role in regulating early developmental processes [2]. These signaling molecules act at low concentrations to coordinate germination dynamics, cell division and elongation, and the establishment of root architecture, while also mediating adaptive responses to environmental stress [4]. Owing to their regulatory capacity, phytohormones are widely applied exogenously through approaches such as seed priming, soil application, and early-stage treatments, with the aim of enhancing seedling vigor, improving establishment success, and promoting early growth under suboptimal conditions [5,6,7].

Despite their potential, the application of free phytohormones remains limited by significant technical and environmental constraints [8]. Their effectiveness is strongly influenced by their stability and availability within the immediate microenvironment surrounding the seed or emerging seedling. Free molecules are prone to rapid degradation, leaching, and poor retention in the rhizosphere, which restricts their effective interaction with plant tissues [4]. Moreover, the precise regulation of hormone concentration is particularly critical during early development, where small variations in endogenous levels can lead to pronounced effects on root elongation, seedling morphology, and overall developmental balance [9,10]. These limitations highlight the need for delivery strategies capable of maintaining controlled and sustained hormone availability during this highly sensitive developmental period [11].

In response to these challenges, hydrogel-based delivery systems have emerged as promising platforms for the encapsulation and controlled release of phytohormones [12,13,14,15]. Hydrogels are three-dimensional polymeric networks capable of retaining large amounts of water, thereby creating a localized hydrated microenvironment around the seed and developing root system [16]. This property not only protects encapsulated molecules from premature degradation but also enables their sustained and regulated release over time [17]. As a result, hydrogel-mediated delivery can enhance the temporal availability of phytohormones in the rhizosphere and improve their interaction with plant tissues during germination and early establishment [18,19]. Additionally, the intrinsic physicochemical properties of hydrogels can improve soil water retention and facilitate root–soil contact, both of which are essential for successful early plant development [20].

Although the beneficial effects of hydrogel-mediated delivery have been widely reported at the level of seedling performance and early growth, the factors underlying their enhanced efficacy remain insufficiently understood. Current evidence suggests that improvements in germination, root development, and stress tolerance are largely associated with enhanced phytohormone stability, retention, and controlled release. This distinction is especially relevant given that encapsulation can modify the temporal availability and effective concentration of signaling molecules within highly sensitive developmental contexts [12,14].

In this context, the present review focuses on plant-derived phytohormones acting during germination, seedling establishment, and early root development, and provides an analysis of their application in hydrogel-encapsulated forms. The central objective is to synthesize current knowledge regarding hydrogel matrices, controlled-release behavior, phytohormone bioavailability, and their reported effects on early plant development. By linking phytohormone biology with hydrogel-mediated delivery strategies, this work aims to provide a critical framework for understanding how controlled release influences phytohormone bioavailability and plant responses, thereby supporting the rational design of more precise, efficient, and sustainable phytohormone-based formulations for early-stage crop development.

## 2. Functional Role of Hydrogels as Plant Substrates in Early Development

Before addressing whether hydrogel-based systems can influence phytohormone availability and early plant development, it is essential to first understand the nature of the hydrogel–plant interface during early development. At the stages of seed germination, seedling establishment, and initial root growth, plant performance is highly dependent on the immediate physicochemical environment surrounding the seed and emerging root system [20]. In this context, hydrogels act as three-dimensional polymeric matrices that position themselves in direct contact with plant tissues, where they retain water, regulate local moisture conditions, and serve as reservoirs for the gradual release of bioactive compounds [17,19]. This interface is not passive; rather, it represents a dynamic zone of interaction between the material, the soil environment, and the developing plant. The transition of hydrogels from simple water-retaining agents to functional materials capable of interacting with roots, soil components, and bioactive molecules highlights their relevance in modern agricultural systems [20,21].

The establishment of this interface is largely determined by the method of application, which defines how the hydrogel interacts spatially and temporally with the plant. Among the different strategies, seed coating and seed priming represent the most relevant approaches for early plant development, as they ensure immediate proximity between the hydrogel matrix and the germinating embryo [18,22,23]. In these configurations, the hydrogel forms a hydrated layer that enhances water uptake, reduces desiccation, and enables the localized release of encapsulated compounds during germination [23]. In addition, hydrogels can be incorporated into the root zone, where they act as reservoirs that release water and bioactive compounds directly into the rhizosphere, or they can be used as partial or total substrates in soil-less systems, providing both physical support and controlled hydration for developing roots [20]. These application strategies are particularly effective because they target the critical interval in which plant establishment is most sensitive to environmental conditions [24].

Beyond their role as delivery matrices, hydrogels function as functional substrates that actively modify the microenvironment surrounding the seed and root system [25]. Their high-water retention capacity allows them to maintain stable moisture conditions, buffering against fluctuations in water availability that are common in soil systems [26,27]. This stabilization is especially important during germination, where water availability directly controls metabolic activation and radicle emergence [28]. At the same time, the porous structure of hydrogels facilitates oxygen diffusion and improves root–substrate contact, enhancing both respiration and nutrient uptake [20]. By interacting with soil particles or acting as independent substrates, hydrogels can modify porosity, water distribution, and aggregation, thereby influencing processes such as water absorption, root elongation, and early plant growth. These combined effects position hydrogels not only as carriers but as active modulators of the rhizosphere environment [16,26].

The effectiveness of these functions is closely linked to the physicochemical properties of the polymers used to construct the hydrogel network. Natural polymers such as alginate, chitosan, cellulose, starch, and gellan gum are widely employed due to their biodegradability, biocompatibility, and environmental compatibility [14,21,29]. These materials form crosslinked networks through ionic interactions, hydrogen bonding, or physical entanglements, resulting in hydrogels with high water affinity and tunable swelling behavior [29]. Beyond their structural role, biodegradation can generate soluble oligomeric fragments capable of interacting with plant tissues and rhizosphere microorganisms [30]. Chitosan-derived oligomers have been associated with the modulation of germination, root development, antioxidant activity, defense signaling, and plant–microbe interactions. Importantly, recent evidence indicates that the biological activity of chitosan is strongly dependent on structural characteristics such as molecular weight, degree of acetylation, concentration, and molecular dispersity, resulting in responses that may range from stimulatory to inhibitory depending on the system evaluated [31].

Synthetic polymers, including polyacrylates, polyethylene glycol, polyvinyl alcohol, and polyacrylamide, are also widely used because they can improve mechanical strength, structural stability, and control over swelling and retention properties [29,32]. However, in agricultural applications, these advantages must be weighed against concerns associated with the limited biodegradability and long-term persistence of some synthetic polymer networks in soil [29]. This consideration is particularly important for materials intended for repeated or large-scale field application, where polymer residues or degradation by-products may accumulate over time [33].

The combination of natural and synthetic polymers in hybrid hydrogels has emerged as a key strategy to optimize performance, enabling fine control over properties such as swelling behavior, mechanical resistance, degradation rate, and diffusion characteristics while balancing functionality and environmental compatibility [20]. This tunability is essential for tailoring the interaction between the hydrogel and the plant system, as it directly influences how water and compounds are retained and distributed in proximity to the root. As a result, the design of the polymeric network becomes a determining factor in the functionality of hydrogels as plant substrates, linking material properties with plant-level responses during early development and establishing the basis for their subsequent role as controlled delivery systems [20,21].

## 3. Hydrogels as Bioactive Delivery Systems and Growth Modulators in Early Plant Development

Building upon the role of hydrogels as functional substrates that define the physicochemical environment of the rhizosphere, their capacity to act as controlled delivery systems becomes critical for regulating bioactive availability during early plant development [1]. At this stage, plant responses are highly sensitive to both the concentration and temporal exposure of signaling molecules, making the regulation of bioactive availability a determining factor in germination efficiency, root establishment, and early growth performance [23]. In this context, hydrogel matrices provide a means to transition from passive environmental modulation to active control over the presence and persistence of bioactive compounds in the immediate microenvironment of the developing plant [16,34].

**Figure 1:**
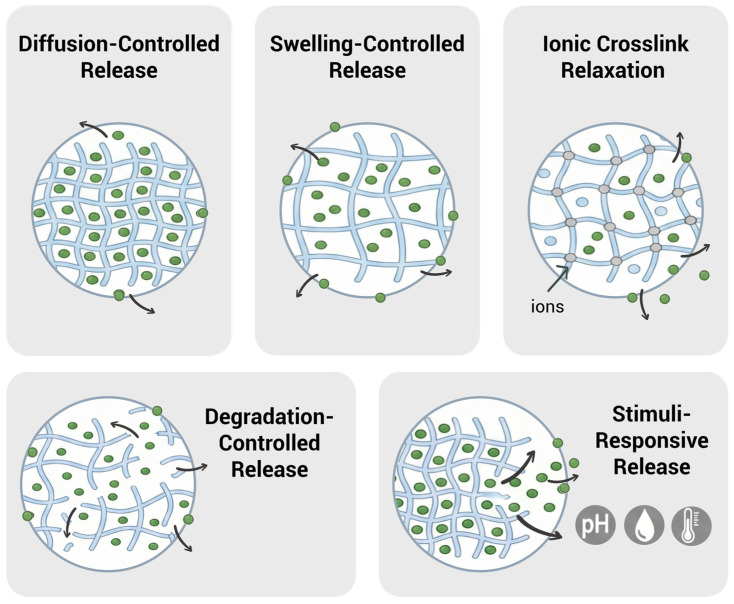
Schematic representation of the mechanisms governing the release of bioactive compounds from hydrogel matrices.

The release of encapsulated bioactive compounds from hydrogels is controlled by the interaction between water uptake, polymer network structure, cargo mobility, and the physicochemical environment surrounding the material [35]. In hydrated networks, water penetration promotes swelling and expands the available free volume between polymer chains, creating pathways for molecular transport. Under these conditions, the mesh size of the network becomes a key parameter because it determines whether the encapsulated molecule can diffuse freely, diffuse under steric restriction, or remain physically retained until additional structural changes occur. Therefore, release is closely related to the relationship between the hydrodynamic size of the phytohormone, the mesh size of the hydrogel, and the degree of crosslinking of the polymeric network. However, diffusion through the hydrated matrix represents only one component of a broader set of release processes, as transport behavior may also be influenced by polymer relaxation, matrix degradation, erosion phenomena, and environmentally responsive structural changes (Figure 1) [35,36,37].

When the encapsulated phytohormone is substantially smaller than the hydrogel mesh size and has limited affinity for the polymer chains, release can occur predominantly through diffusion across water-filled domains of the swollen network. In this case, the concentration gradient between the hydrogel and the surrounding rhizosphere acts as the main driving force for transport, and the release profile may be reasonably approximated using Fickian diffusion [17]. However, this description is most appropriate for relatively stable networks in which swelling reaches equilibrium rapidly and the polymeric matrix does not undergo significant relaxation, degradation, or chemical reorganization during the release period. As a result, purely Fickian transport is generally restricted to systems in which structural changes within the hydrogel occur at a much slower rate than molecular diffusion [35,38].

In many hydrogel systems, particularly those with high swelling capacity, release cannot be interpreted only as diffusion through a static network. As water enters the matrix, polymer chains progressively relax, the mesh size changes, and the internal transport pathways evolve over time. Under these conditions, solute diffusion and polymer relaxation occur simultaneously, giving rise to anomalous or non-Fickian transport. This behavior is especially relevant for hydrogels whose swelling kinetics are comparable to the rate of cargo diffusion, because the release profile is then determined by both molecular mobility and time-dependent structural rearrangement of the network [35,38].

The crosslinking mechanism further modifies release behavior. Ionically crosslinked polysaccharide hydrogels, such as alginate-, chitosan-, or gellan-based systems, are particularly sensitive to pH, ionic strength, and ion exchange in the surrounding medium [29]. Changes in the ionic composition of the soil solution can weaken or rearrange ionic junctions, modify swelling capacity, and alter network permeability, thereby accelerating or delaying phytohormone release [19]. In contrast, covalently crosslinked networks generally exhibit greater structural persistence and may sustain release through slower diffusion across a more stable matrix [17]. Thus, differences in crosslinking chemistry can produce distinct release profiles even for systems designed to deliver similar bioactive compounds.

Biodegradable hydrogels introduce an additional release pathway associated with the progressive degradation of the polymer matrix. Unlike diffusion-controlled systems, where transport occurs primarily through pre-existing water-filled pathways, degradation-mediated release depends on structural modifications of the network itself [36]. Hydrolytic cleavage of polymer chains, disruption of labile crosslinks, enzymatic degradation, and surface erosion can progressively increase mesh size and network permeability, facilitating the release of phytohormones that remain partially or completely entrapped within the matrix. The relative contribution of these processes depends on polymer composition, crosslinking chemistry, environmental conditions, and the susceptibility of the material to biological degradation. Consequently, release may occur not only as a result of molecular diffusion but also through the gradual loss of structural integrity of the hydrogel [35,36]. This mechanism is particularly relevant for agricultural applications because biodegradable matrices are continuously exposed to microbial activity, extracellular enzymes, dissolved ions, fluctuations in soil moisture, and repeated wetting–drying cycles, all of which can accelerate matrix degradation and modify release kinetics [29]. As a result, hydrogel performance in soil is frequently governed by the combined action of diffusion, swelling, degradation, and erosion processes rather than by a single transport mechanism operating in isolation.

Stimuli-responsive hydrogels represent a further level of control because their release behavior is intentionally coupled to environmental triggers [13]. Variations in pH, moisture, ionic strength, temperature, redox conditions, enzymatic activity, or chemical signals can modify the swelling state, charge density, crosslink stability, or degradation rate of the polymer network [39]. As a result, phytohormone release is no longer governed solely by concentration gradients or matrix permeability, but also by external stimuli capable of altering the physicochemical properties of the hydrogel. These systems are therefore better described as environmentally regulated delivery platforms rather than passive diffusion reservoirs [39]. Such behavior is particularly attractive for agricultural applications because the rhizosphere is a highly dynamic environment in which root exudation, microbial metabolism, nutrient availability, and soil moisture continuously fluctuate [40]. Consequently, stimuli-responsive hydrogels offer the possibility of synchronizing phytohormone release with local environmental conditions, potentially improving delivery efficiency while reducing unnecessary losses associated with continuous or uncontrolled release.

The controlled release provided by hydrogels directly influences the subsequent transport and uptake of bioactive compounds within the plant system. Once released into the rhizosphere, these molecules establish localized concentration gradients that drive their movement toward the root surface through diffusion and mass flow [41]. Their proximity to the root interface enhances the probability of uptake, which occurs primarily through epidermal and cortical tissues [20]. Following absorption, bioactive compounds are transported within the plant via apoplastic and symplastic pathways, enabling their movement through cell walls, intercellular spaces, and cytoplasmic connections toward the vascular system [4,42]. This internal distribution allows for the systemic delivery of signaling molecules to actively grow tissues, linking localized release with whole-plant responses. Importantly, the efficiency of this process is not only dependent on the properties of the compounds themselves but also on the spatial and temporal patterns of release established by the hydrogel matrix [4,20,43].

**Figure 2:**
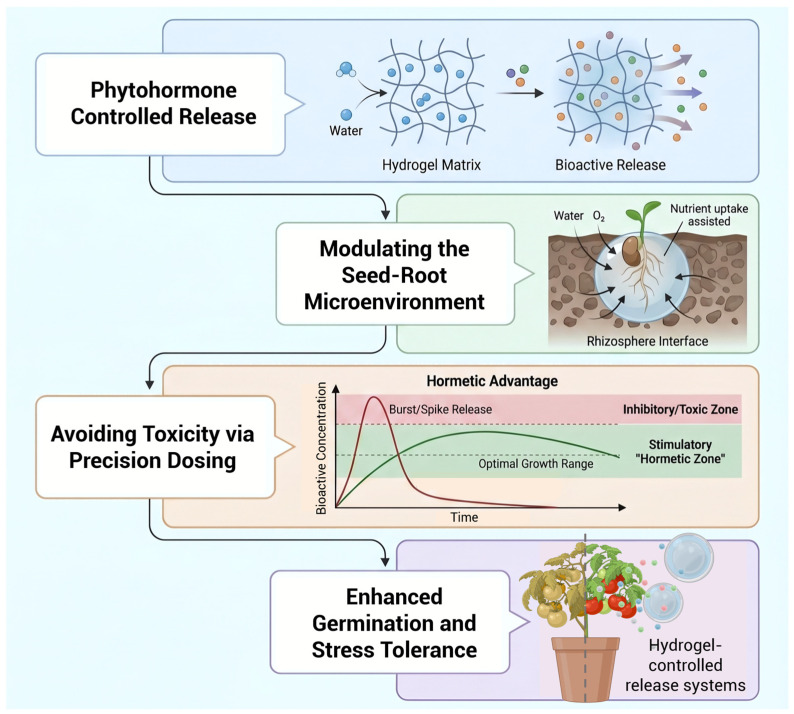
Swelling and diffusion-driven release from hydrogels regulates bioactive levels at the seed-root interface, regulating bioactive availability at the seed–root interface through controlled release, enhancing germination and stress tolerance.

In this context, hydrogels can be understood as effective growth modulators, not merely due to their influence on the physical environment, but because of their ability to regulate the exposure of plants to bioactive compounds [20]. This function is particularly relevant in the context of hormesis, a dose-dependent response characteristic of many plant-derived bioactives. Under hormetic behavior, low concentrations of a compound stimulate physiological processes such as cell division, elongation, and root development, whereas higher concentrations may lead to inhibitory or toxic effects [4]. However, hormetic responses are highly dependent on the specific phytohormone, plant species, developmental stage, target tissue, and environmental conditions [44]. Rather than maintaining a fixed hormetic threshold, hydrogel-based delivery systems aim to reduce abrupt concentration fluctuations and provide sustained exposure profiles that can be adjusted to the physiological requirements of specific crops and developmental stages [45]. In this context, controlled-release formulations may reduce the risk of phytotoxicity associated with uncontrolled hormone exposure while improving the temporal availability of bioactive compounds within the rhizosphere (Figure 2) [4].

This controlled delivery contributes to the mitigation of both abiotic and biotic stresses, which are particularly critical during early plant development [1,46]. Under abiotic stress conditions such as drought, salinity, and temperature fluctuations, hydrogels contribute not only by improving water availability but also by enabling the sustained release of compounds that support physiological adaptation. These include molecules that enhance osmotic regulation, stabilize cellular structures, and reduce oxidative damage through the modulation of antioxidant systems [15,18]. Similarly, in the context of biotic stress, hydrogel-based delivery systems facilitate the continuous supply of compounds associated with plant defense, contributing to the activation of protective responses and the reduction of pathogen-induced damage [4,47]. By maintaining a consistent presence of these bioactives, hydrogels enhance plant responsiveness, improving their capacity to respond to environmental challenges during vulnerable developmental stages.

Among the diverse range of compounds that can be delivered through hydrogel systems, plant-derived phytohormones play a central role due to their function as key regulators of plant growth and stress responses [18,48]. Molecules such as auxins (e.g., indole-3-acetic acid (IAA)), gibberellins (GAs), cytokinins (CKs), abscisic acid (ABA), salicylic acid (SA), jasmonic acid (JA), brassinosteroids (BRs), strigolactones (SLs), karrikins (KARs), polyamines, and melatonin (Mel) are involved in processes ranging from germination and root development to stress signaling and defense regulation [2]. Given that plant development relies on tightly controlled hormonal balance, the ability of hydrogels to regulate the timing, concentration, and localization of these signals represents a significant advantage. By preventing abrupt fluctuations and ensuring sustained availability, hydrogel-mediated delivery systems enable a more stable and controlled hormonal environment during early development [11,18].

The studies summarized in Table 1 illustrate the considerable variability in release behavior among hydrogel-based phytohormone delivery systems. Release durations ranged from hours to several weeks, depending on hydrogel composition, release conditions, and the physicochemical properties of the encapsulated phytohormone. For example, poly(lactic-co-glycolic acid)-poly(ethylene glycol)-poly(lactic-co-glycolic acid) (PLGA-PEG-PLGA) hydrogels loaded with SA exhibited relatively rapid release profiles, with cumulative release values of 1.5–14% over periods shorter than 24 h [15]. In contrast, polysaccharide-based matrices such as sodium alginate-carboxymethyl cellulose (SA-CMC) and alginate/chitosan/dopamine hydrogels sustained the release of naphthalene acetic acid (NAA) and GA for 15–21 days while achieving cumulative release values exceeding 75%. Differences were also observed in the underlying release mechanisms, which ranged from diffusion-controlled transport to anomalous release involving both diffusion and polymer relaxation [12,14]. Furthermore, cellulose/polyvinyl alcohol (PVA) hydrogels demonstrated a strong dependence on environmental conditions, with cumulative SA release varying from 31.0% to 90.4% as a function of pH and redox state [13]. Collectively, these findings highlight the importance of hydrogel composition, network architecture, and environmental responsiveness in determining phytohormone availability and release performance.

Differences in release duration, cumulative release, and transport mechanisms demonstrate that hydrogel composition can substantially influence the spatiotemporal distribution of phytohormones within the rhizosphere. Because phytohormones regulate plant development through highly dynamic and interconnected signaling networks, variations in hormone exposure may affect not only the magnitude of individual responses but also the coordination of developmental and stress-related processes [8]. Consequently, the biological effects associated with hydrogel-mediated delivery cannot be explained solely by enhanced hormone retention or prolonged release, but must also be considered in the context of how changes in phytohormone dynamics influence plant signaling pathways and developmental regulation.

## 4. Interplay Between Hydrogel-Mediated Delivery and Phytohormone Signaling in Early Plant Development

Building on the role of hydrogels as platforms capable of regulating the spatial and temporal availability of bioactive compounds, it is essential to examine how this controlled delivery influences phytohormone performance during early plant development [16]. At the stages of germination and seedling establishment, plant responses are governed by tightly coordinated hormonal networks in which concentration thresholds, spatial gradients, and exposure time determine developmental outcomes. These processes are highly sensitive to fluctuations in hormone availability, making the regulation of phytohormone exposure particularly important during early growth [4,11]. Conventional exogenous application frequently leads to rapid degradation, uncontrolled diffusion, and transient concentration peaks that can disrupt endogenous hormonal balance [49,50]. In contrast, hydrogel-based systems provide a means to regulate hormone availability over time, potentially improving the effectiveness of phytohormone-based treatments during critical developmental stages. However, because plant development depends on dynamic and context-dependent hormonal interactions, the biological consequences of controlled release must be considered not only in terms of hormone persistence, but also in relation to the temporal requirements of specific signaling pathways [12,14].

IAA is a heterocyclic compound containing a carboxymethyl group (acetic acid) that belongs to the auxin phytohormone family and regulates multiple processes in plant growth and development under both normal and environmental stress conditions [51]. IAA regulates early plant development through polar auxin transport and receptor-mediated signaling involving the TIR1/AFB complex, which promotes degradation of Aux/IAA repressors and activation of auxin response factors (ARF). The establishment of auxin gradients, mediated by efflux (pin-formed, PIN) carriers and influx (auxin transporter carrier1/auxin transporter-like proteins, AUX1) proteins, is essential for root patterning and gravitropic responses, and is highly sensitive to fluctuations in local concentration [11,52]. Under conventional application, free IAA often leads to transient concentration spikes that disrupt these gradients, resulting in inhibited primary root elongation and altered root architecture [4,42]. Controlled-release systems demonstrate that these limitations can be mitigated through encapsulation strategies. For instance, auxin-loaded polymeric hydrogels enhanced rooting initiation and elongation compared with conventional treatments [53,54,55]. Importantly, the effectiveness of auxin delivery depends not only on maintaining hormone availability but also on preserving the gradients required for normal developmental signaling.

GAs are a class of tetracyclic diterpenoid phytohormones involved in a wide range of plant growth and developmental processes, including seed germination, stem elongation, flowering, fruit development, and senescence-related processes [56]. GA is essential for breaking seed dormancy and mobilizing endosperm reserves, directly fueling radicle protrusion and subsequent hypocotyl elongation [57,58]. GA signaling involves the binding of the hormone to the GID1 receptor, which initiates the ubiquitination and proteasomal degradation of DELLA repressor proteins, and activates growth-related gene expression, including enzymes responsible for reserve mobilization, such as α-amylase [4]. Importantly, GA activity is tightly coordinated with other hormonal pathways, particularly ABA signaling, which maintains seed dormancy and opposes germination-related processes [56]. Consequently, successful germination depends not only on GA availability but also on the temporal balance between antagonistic hormonal signals. Free GA application is highly susceptible to rapid environmental leaching and often triggers excessively rapid, fragile cell elongation that compromises seedling structural integrity [58]. Chitosan/alginate hydrogel systems have been shown to successfully encapsulate GA, significantly enhancing tomato and bean seed germination, photosynthetic efficiency, and drought tolerance [14,58,59,60].

CKs are adenine-derived phytohormones essential for plant growth and development, particularly in regulating cell division, differentiation, and the delay of leaf senescence. They are characterized by a side chain at the N^6^-position, which can be either isoprenoid (e.g., zeatin) or aromatic (e.g., benzyladenine) [61]. Their signaling occurs via a multi-step phosphorelay system involving histidine kinase receptors, phosphotransfer proteins, and response regulators that coordinate developmental programs in response to hormonal and environmental cues [61]. A defining characteristic of CK function is its close interaction with auxin signaling, as the balance between these two phytohormones plays a central role in regulating meristem activity, root patterning, and organ development [61]. Consequently, developmental outcomes are influenced not only by the absolute concentration of CKs but also by their temporal and spatial relationship with auxin gradients. Under conventional application, CKs are limited by rapid enzymatic cleavage by cytokinin oxidases and the potential to overly suppress root elongation when applied in unbuffered doses [62]. Although hydrogel-based delivery of CKs remains relatively unexplored, controlled-release systems may improve hormone persistence and reduce abrupt concentration fluctuations.

ABA is a sesquiterpenoid phytohormone with a central role in plant development and stress responses. Initially associated with leaf abscission, ABA accumulates in response to environmental stresses such as low temperature, dehydration, and photoperiod changes. It regulates seed dormancy, inhibition of precocious germination, and the accumulation of storage compounds during embryo development [4,63]. During early plant development, ABA acts as a key integrator of environmental and endogenous signals, controlling the balance between dormancy and germination. Its perception through PYR/PYL/RCAR receptors initiates a kinase cascade involving the inhibition of PP2C phosphatases and activation of SnRK2 kinases, which is highly sensitive to fluctuations in environmental conditions [2]. Importantly, ABA functions in close antagonism with GA during germination, and successful seedling establishment depends on the coordinated transition from ABA-dominated dormancy signaling to GA-mediated growth promotion [2]. Under conventional application, ABA is limited by its photosensitivity, as ultraviolet radiation can induce conformational changes that reduce its biological activity, as well as by its tendency to inhibit growth when applied at elevated concentrations [63]. Encapsulation strategies, such as alkali lignin–cetyltrimethylammonium bromide (AL–CTAB) nanomicroparticles, have demonstrated improved photostability and more sustained release into the rhizosphere [63]. Such controlled delivery may enhance hormone persistence and reduce abrupt concentration fluctuations. Future hydrogel-based delivery systems should consider not only sustained ABA availability but also the dynamic hormonal interactions that govern dormancy release and early plant development.

SA is a phenolic phytohormone, synthesized from the shikimic acid pathway, essential for plant growth, development, and immune responses, particularly in mediating defense against biotrophic pathogens and environmental stress [64]. SA signaling is mediated through the NPR1 regulatory complex and is closely associated with redox balance and transcriptional activation of defense-related genes. It also interacts with auxin transport pathways, contributing to the regulation of growth–defense trade-offs. This interaction is characterized by a hormetic response, in which moderate concentrations enhance antioxidant activity, whereas elevated levels can disrupt auxin homeostasis, restrict cell elongation, and impair root development [64,65,66]. The uncontrolled release of free SA can shift this balance toward inhibitory responses. Encapsulation strategies, including silica- and chitosan-based carriers as well as dual-responsive bagasse cellulose/PVA hydrogel systems, enable more gradual and sustained hormone delivery [13,46,49]. Such controlled release profiles may help maintain SA availability within physiologically relevant ranges.

JA and its derivatives are lipid-derived signaling compounds (oxylipins) involved in plant growth, development, and immune responses. Their biological activity is modulated by structural modifications such as amino acid conjugation (e.g., jasmonate-isoleucine (JA-Ile)) and methylation (MeJA) [50]. JA and its volatile derivative MeJA function as central regulators of plant defense against necrotrophic pathogens and herbivores, while also contributing to responses to abiotic stresses such as cold and salinity [67]. The signaling cascade is mediated through SCF^COI1^-dependent degradation of JAZ repressor proteins, which releases MYC transcription factors and activates defense-related gene expression [2]. In addition to its protective role, JA participates in extensive hormonal crosstalk with growth-regulating pathways, including auxin and gibberellin signaling. As a result, activation of JA-dependent defense responses is frequently associated with growth inhibition, reflecting a physiological trade-off that allows plants to prioritize survival under adverse conditions [50]. In practical applications, JA and MeJA are limited by rapid environmental dissipation, high volatility, and pronounced growth–defense trade-offs, where strong activation of defense pathways can suppress early plant growth [68]. Encapsulation strategies, such as MeJA-loaded chitosan nanoparticles, enable more controlled and sustained release profiles [69]. While these systems may enhance stress tolerance and antioxidant responses, the biological consequences of sustained JA exposure must be interpreted carefully because prolonged activation of defense pathways may not always be compatible with the growth requirements of developing seedlings [68].

BRs are polyhydroxylated steroidal phytohormones that regulate cell expansion, vascular differentiation, and photomorphogenesis, often acting in coordination with auxins to support adaptation to abiotic stresses such as drought and temperature fluctuations. Upon binding to the plasma membrane receptor kinase BRI1, BRs initiate a signaling cascade that relieves repression of downstream components, leading to transcriptional reprogramming and modulation of reactive oxygen species (ROS) homeostasis [70]. Practical applications of BRs are often limited by rapid metabolic turnover in plant tissues and low environmental persistence [4]. Although hydrogel-based delivery of BRs remains relatively unexplored, insights from controlled-release systems for other bioactive compounds provide a basis for evaluating their potential behavior [12]. Because BR-mediated responses depend on interactions with multiple hormonal pathways, future studies should evaluate not only hormone persistence but also how delivery profiles influence the coordination of BR signaling with other developmental and stress-related regulatory networks [70].

SLs are carotenoid-derived phytohormones that regulate root architecture, suppress shoot branching, and function as rhizosphere signals that facilitate symbiotic interactions with arbuscular mycorrhizal fungi [4,71]. SL perception is mediated by the α/β-hydrolase receptor D14, which, upon ligand binding, interacts with the F-box protein MAX2, promoting ubiquitination and degradation of SMXL/D53 repressors. This process activates downstream transcriptional programs that modulate shoot branching, root hair development, and auxin transport through effects on PIN protein localization and abundance. During early development, SLs contribute to the regulation of root system architecture and nutrient acquisition, partly through their interaction with auxin transport and signaling pathways [72,73]. This relationship highlights the importance of coordinated hormonal regulation, as changes in SL availability can influence auxin distribution and consequently affect root development and plant plasticity under nutrient-limited conditions. The practical application of SLs is constrained by their chemical instability in soil and rapid hydrolysis following exogenous application [73]. Although direct hydrogel-based studies remain limited, encapsulation within polymeric matrices may improve local retention and protect SLs from premature degradation.

KARs are smoke-derived butenolide compounds that trigger seed germination and seedling photomorphogenesis, particularly in dormant seeds and post-fire ecological contexts [74,75]. They are perceived by the receptor KAI2, which interacts with the F-box protein MAX2, promoting the degradation of repressors such as SMAX1/SMXL2 and activating transcriptional programs associated with germination and early development. KAR signaling is especially relevant during early stages, where it modulates seed sensitivity to environmental cues such as light and moisture. Despite their high biological activity at low concentrations, KARs are susceptible to environmental loss, which limits their effectiveness under field conditions [74,75,76]. Controlled-release systems may offer a potential strategy to improve local retention within the seed microenvironment. However, because KAR-mediated responses are closely associated with specific developmental transitions, future delivery systems should consider not only maintaining hormone availability but also synchronizing release with the temporal requirements of germination and early seedling establishment [74,75,76,77].

Polyamines such as spermidine, spermine, and putrescine are low-molecular-weight aliphatic polycations that regulate cell cycle progression, membrane stability, and stress responses during early plant development [78]. Their biosynthesis is linked to amino acid metabolism through arginine and ornithine pathways, and their activity arises from electrostatic interactions with nucleic acids, proteins, and membranes, as well as modulation of ion channels and signaling cascades [24,28,79]. Polyamines are closely associated with ROS homeostasis, acting both as direct scavengers and as regulators of antioxidant enzymes such as superoxide dismutase (SOD), catalase (CAT), and ascorbate peroxidase (APX) [79,80]. In addition, they interact with hormonal pathways, including auxin and ABA signaling, influencing root development and stress tolerance [81]. Exogenous application of free polyamine treatments is highly concentration- and species-dependent. While beneficial at appropriate levels, burst application or imbalanced ratios can exacerbate stress responses, partly through excessive apoplastic hydrogen peroxide production. Furthermore, rapid oxidation by amine oxidases limits their persistence when applied in free form [28,79,82]. Polyamines have also been incorporated into hydrogel systems not only as encapsulated bioactives but as structural components, for example, as cationic crosslinkers in polysaccharide matrices such as gellan gum [29]. In these systems, gradual matrix degradation may simultaneously influence hydrogel properties and local polyamine availability [29]. Consequently, hydrogel-mediated delivery provides an interesting framework for regulating polyamine exposure while potentially contributing to more stable physiological responses during early plant establishment.

Mel is a naturally occurring low molecular weight indoleamine compound involved in growth modulation, stress tolerance, and redox signaling. It is synthesized from tryptophan via serotonin intermediates and exerts its effects through both direct antioxidant activity and regulation of gene expression [6,83]. Mel enhances the activity of antioxidant enzymes such as SOD, CAT, and peroxidases, while also influencing mitochondrial function and maintaining cellular redox balance. During early development, Mel supports seed germination, root growth, and stress responses by integrating ROS signaling with hormonal crosstalk, particularly with auxin and ABA pathways. However, Mel is highly susceptible to degradation and rapid metabolism, limiting its persistence when applied exogenously [6,83]. Although hydrogel-based delivery studies remain limited, controlled-release systems may provide a strategy to improve local retention and prolong Mel availability within the rhizosphere. Future studies should evaluate how different release profiles influence not only Mel persistence but also its coordination with the signaling networks that regulate early plant development [6].

**Figure 3:**
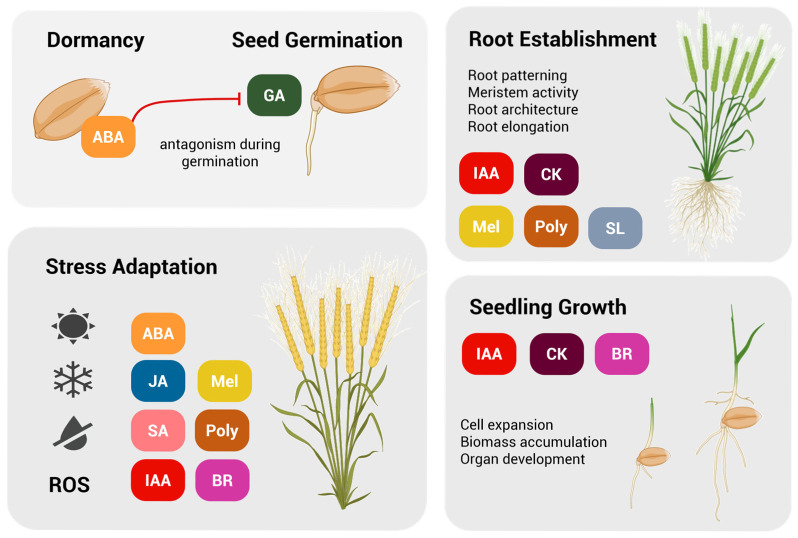
Representative phytohormonal interactions associated with seed germination, root establishment, seedling growth, and abiotic stress adaptation.

Taken together, these observations demonstrate that the biological effects of hydrogel-mediated phytohormone delivery extend beyond simple improvements in hormone retention or persistence within the rhizosphere. The physiological outcomes associated with controlled-release systems are ultimately determined by the specific functions of each phytohormone, their concentration-dependent effects, and their interactions with other signaling pathways involved in plant growth, development, and stress adaptation. Consequently, the effectiveness of hydrogel-mediated delivery should be evaluated not only in terms of hormone availability but also in relation to the hormonal networks that regulate developmental responses during germination, seedling establishment, and early plant growth (Figure 3).

In this context, hydrogels can be regarded as advanced delivery platforms that regulate phytohormone availability during critical stages of plant development. By influencing the timing, localization, and duration of hormone exposure, these systems can modulate physiological processes associated with germination, root establishment, stress adaptation, and early seedling growth. Representative examples of hydrogel-mediated phytohormone delivery systems, their evaluation models, and the biological responses reported are summarized in Table 2.

## 5. Conclusions and Future Perspectives

In synthesizing the current understanding of hydrogel-mediated phytohormone delivery, it becomes evident that the primary contribution of hydrogel systems lies not in altering the intrinsic biological functions of phytohormones but in regulating the conditions under which these signaling molecules become available to plants. By controlling the timing, localization, and duration of hormone exposure, hydrogels can improve germination, seedling establishment, root development, and stress adaptation during critical stages of plant development. These benefits are generally associated with improved phytohormone bioavailability and more controlled delivery profiles rather than with direct modification of the underlying signaling pathways.

Despite these advances, important limitations remain. Most studies report phenotypic and physiological outcomes (germination rate, root length, and biomass), whereas direct molecular evidence explaining how controlled-release systems influence phytohormone signaling remains scarce. Critical questions that require further investigation include (i) how hydrogel-mediated release dynamics influence phytohormone perception and receptor activation; (ii) how controlled delivery affects transcriptional regulation, hormonal crosstalk, and downstream signaling networks; (iii) how hydrogel degradation, soil chemistry, and rhizosphere microbiomes collectively modify phytohormone bioavailability under realistic agricultural conditions; and (iv) whether the biological benefits observed under laboratory and greenhouse conditions can be consistently reproduced under heterogeneous field environments. Addressing these challenges will require the integration of transcriptomic, proteomic, metabolomic, and time-resolved analytical approaches together with long-term field validation studies. Such efforts will be essential for establishing the mechanistic basis of hydrogel-mediated phytohormone delivery and for guiding the development of more effective and predictable controlled-release technologies.

Future developments should move beyond the objective of simply prolonging phytohormone release. Plant development is governed by highly dynamic hormonal networks in which signaling molecules frequently act sequentially, interact antagonistically or synergistically, and exhibit stage-dependent biological functions. Consequently, sustained release may not always represent the optimal delivery strategy for all phytohormones or developmental processes. Advanced hydrogel systems capable of providing sequential, stage-specific, or environmentally triggered release profiles, including multilayer architectures, differential degradation strategies, and stimuli-responsive matrices, represent promising directions for improving delivery precision and better aligning phytohormone availability with plant developmental requirements. The integration of advanced materials engineering, nanotechnology, and bioengineering approaches may further enhance loading capacity, cargo protection, and delivery precision while enabling more sophisticated control over phytohormone release under realistic agricultural conditions.

From an agronomic perspective, hydrogel-mediated phytohormone delivery systems offer the potential to improve the efficiency of hormone-based treatments. However, successful implementation at commercial scale will depend not only on biological performance but also on economic feasibility, compatibility with existing seed coating, priming, and sowing technologies, and long-term environmental safety. Although improvements in germination, establishment success, stress tolerance, and early growth have been widely reported, the costs associated with hydrogel synthesis, phytohormone encapsulation, quality control, storage, and large-scale deployment remain important considerations. Future studies should therefore incorporate techno-economic analyses and field-scale evaluations to determine the practical feasibility and cost–benefit relationships of hydrogel-based delivery systems across different cropping systems and environmental conditions.

Overall, hydrogel-mediated phytohormone delivery represents a promising strategy for improving plant establishment and resilience through the precise regulation of hormone availability during early development. Continued progress will depend on integrating advances in materials science, plant physiology, molecular biology, and agronomic validation to develop delivery systems that are biologically effective, environmentally sustainable, economically viable, and applicable under real agricultural conditions. Ultimately, the ability to regulate phytohormone availability in space and time may provide new opportunities for improving crop establishment and productivity in increasingly challenging agricultural environments.

## Figures and Tables

**Table 1:** Summary of hydrogel-mediated phytohormone delivery systems and their release characteristics.

Hydrogel Matrix	Phytohormone Released	Initial Concentration	Release Medium	Cumulative Release (%)	Release Time	Release Mechanism	Source
PLGA-PEG-PLGA (Triblock copolymer)	SA	20 mg/mL	Phosphate-buffered saline	14	17.5 h	Not reported	[15]
PLGA-PEG-PLGA (Triblock copolymer, 3D printed mesh)			Deionized water	1.5	9.16 h		
SA/CMC	NAA	0.385 mg/mL	Deionized water	77	21 days	Diffusion-controlled release	[12]
Alginate/chitosan/dopamine	GA	20 mg/mL	Phosphate-buffered saline	86.3 (pH 5.0), 73.71 (pH 7.0), 79.1 (pH 9.0)	15 days	Anomalous transport	[14]
Cellulose/PVA	SA	Not reported	Buffer solutions (pH 7.4 and 5.0) with or without 10 mM of reducing agent glutathione (GSH)	31.0 (pH 7.4), 46.8 (pH 5.0), 58.4 (pH 7.4 + GSH), 90.4 (pH 5.0 + GSH)	60 h	Diffusion control and stimulus control release	[13]

**Table 2:** Summary of hydrogel-mediated phytohormone delivery systems, evaluation models, and observed biological effects.

Phytohormone Released	Materials	Evaluation Models	Effect	Source
IAA	Lignin	Tomato (*Solanum lycopersicum* L.)	Lowest concentration of encapsulated IAA increased the number of fruits. At higher concentrations, toxic effects contributed to a reduction in fruit number.	[42]
	V-type starch	Tomato (*Solanum lycopersicum* L.)	Low concentration promotes growth, while high concentration inhibits growth, like free IAA.	[55]
	Chitosan	Malling Merton 106 (MM106) apple rootstock	In vitro adventitious rooting of MM106 microcuttings was higher with delivery systems compared to free hormones.	[54]
	Lignin	Tomato (*Solanum lycopersicum* cv. Sweet Grape) and wheat (*Triticum aestivum*)	Encapsulation enables controlled release and enhanced xylem development in tomato and wheat without phytotoxicity.	[53]
GA	Alginate/chitosan/dopamine	Tomato (*Solanum lycopersicum* L.)	Encapsulation enhances seed germination and plant growth more effectively than free GA.	[14]
	Alginate/chitosan	Cherry tomato (*Solanum lycopersicum* cv. Cerasiforme)	Encapsulation enhances drought tolerance by maintaining growth, photosynthetic activity, and antioxidant responses more effectively than free GA or polymeric treatments alone.	[58]
	Alginate/chitosan	Bean (*Phaseolus vulgaris* L.)	Encapsulation increased leaf area and enhanced chlorophyll and carotenoid levels.	[60]
ABA	Lignin/CTAB	Arabidopsis (*Arabidopsis thaliana*) and rice (*Oryza sativa* L.)	Formulation improves photostability and controlled release, enhancing inhibition of seed germination and drought tolerance more effectively than free ABA.	[63]
SA	Silica/chitosan	*Arabidopsis thaliana*	Encapsulation enables controlled release, reducing negative effects on root and shoot development by preventing rapid uptake, excessive accumulation, and disruption of auxin homeostasis compared to free SA.	[46]
	Cellulose/PVA	Dual-responsive controlled-release was carried out in different pH buffers and concentrations of glutathione solutions.	The release was controlled under different pH conditions and in the presence of reducing agent glutathione.	[13]
	PLGA-PEG-PLGA	Physicochemical evaluation with simulated media-controlled release.	Sustained delivery of SA for plant stress resilience, enhanced adaptability to environmental conditions.	[15]
	Silica/chitosan	*Arabidopsis thaliana*	Encapsulated enhances stress tolerance by maintaining endogenous SA and auxin homeostasis, preserving normal signaling activity, and avoiding overactivation of stress-related gene expression compared to free SA.	[49]
MeJA	Chitosan	Rice (*Oryza sativa* L. *japonica*)	MeJa loading enhances and prolongs enzyme activity and secondary metabolite production, demonstrating sustained elicitor effects compared to non-loaded systems.	[69]
Mel	Alginate	Tomato (*S. lycopersicum* L. cultivar “-Dafni F1”)	Improve tolerance to salinity stress, enhancing agronomic and biochemical responses compared to non-treated systems.	[18]

## Data Availability

No new data were created or analyzed in this study. Data sharing is not applicable to this article.
